# Reprofiled anthelmintics abate hypervirulent stationary-phase *Clostridium difficile*

**DOI:** 10.1038/srep33642

**Published:** 2016-09-16

**Authors:** Major Gooyit, Kim D. Janda

**Affiliations:** 1 Departments of Chemistry and Immunology and Microbial Science, The Skaggs Institute for Chemical Biology, and The Worm Institute of Research and Medicine (WIRM), The Scripps Research Institute, 10550 North Torrey Pines Road, La Jolla, California 92037, United States

## Abstract

Prolonged use of broad-spectrum antibiotics disrupts the indigenous gut microbiota, which consequently enables toxigenic *Clostridium difficile* species to proliferate and cause infection. The burden of *C. difficile* infections was exacerbated with the outbreak of hypervirulent strains that produce copious amounts of enterotoxins and spores. In recent past, membrane-active agents have generated a surge of interest due to their bactericidal property with a low propensity for resistance. In this study, we capitalized on the antimicrobial property and low oral bioavailability of salicylanilide anthelmintics (closantel, rafoxanide, niclosamide, oxyclozanide) to target the gut pathogen. By broth microdilution techniques, we determined the MIC values of the anthelmintics against 16 *C. difficile* isolates of defined PCR-ribotype. The anthelmintics broadly inhibited *C. difficile* growth *in vitro* via a membrane depolarization mechanism. Interestingly, the salicylanilides were bactericidal against logarithmic- and stationary-phase cultures of the BI/NAP1/027 strain 4118. The salicylanilides were poorly active against select gut commensals (*Bacteroides*, *Bifidobacterium* and *Lactobacillus* species), and were non-hemolytic and non-toxic to mammalian cell lines HepG2 and HEK 293T/17 within the range of their *in vitro* MICs and MBCs. The salicylanilide anthelmintics exhibit desirable properties for repositioning as anti-*C. difficile* agents.

*Clostridium difficile* infections (CDI) has plagued nearly half a million Americans that resulted in 29,300 deaths in 2011^1^, and the propensity of nosocomial CDI recurrence has been observed in up to 50% of patients^2^. The growing epidemic of CDI has been largely attributed to the emergence of the hypervirulent strain BI/NAP1/027^3^,^4^,^5^, coupled with the paucity of therapeutics that specifically target the Gram-positive, spore-forming bacillus as well as, prevent the recrudescence of the disease. Although current treatment options (metronidazole and vancomycin) are still able to manage moderate cases of CDI, the escalating rates of fulminant and recurrent infections pose a major threat that warrant immediate attention. Fidaxomicin is a non-absorbed oral macrocyclic antibiotic that was approved by the FDA in 2011 for the treatment of CDI. It demonstrated similar rates of clinical cure as vancomycin^6^,^7^ and significantly lowered the rate of recurrence of non-NAP1-associated infections^6^ –a finding that is attributable to its high selectivity against *C. difficile*^8^,^9^ and its ability to inhibit toxin and spore production in the offending pathogen^10^,^11^. However, there was no difference in outcomes observed for patients that were infected with the hypervirulent BI/NAP1/027 strain^6^. Although resistance is not widespread as of yet, *C. difficile* strains with reduced susceptibility to metronidazole, vancomycin or fidaxomicin have already been noted^12^,^13^,^14^.

The persistence of CDI is alarming in its breadth and points to the pressing need to identify effective treatment options. As a result, the scientific community has risen to the challenge of developing alternative small molecule and biotherapeutic strategies to combat CDI^15^. It is evident that anti-difficile agents with low oral bioavailability (to localize the drug at the site of infection) and a narrow antimicrobial spectrum (to minimize collateral damage to the resident gastrointestinal microbiome) are preferable. Hypervirulent *C. difficile* isolates have been shown to produce toxins (TcdA and TcdB) and spores primarily during the stationary phase of growth^4^. This sets an impediment because quiescent stationary-phase cells are especially resilient to antimicrobial chemotherapy^16^. An emerging strategy to combat refractory *C. difficile* is to target the vulnerability of its membrane. The clinical relevance of this approach lies in the importance of the microbial membrane in both metabolizing and non-growing cells, and the associated cellular effects that could limit the likelihood of bacteria to develop resistance^17^. Indeed, membrane-active agents have demonstrated potential in eliminating stationary-phase *C. difficile* cells, which subsequently led to a substantial decrease in toxin production and sporulation^16^,^18^,^19^.

Salicylanilides have been reported to exhibit antimicrobial properties^20^,^21^ albeit they are chiefly exploited as antiparasitic agents. Closantel (**1**), rafoxanide (**2**), niclosamide (**3**) and oxyclozanide (**4**) represent four of the widely used salicylanilide anthelmintics (Fig. 1). Niclosamide is an FDA-approved drug for the treatment of tapeworm infections, while the other three are marketed as veterinary drugs for liver fluke/roundworm infections in ruminants^22^. The exact antibacterial mode of action of salicylanilides is not well defined but is thought to involve dissipation of the (trans)membrane potential or the proton motive force (pmf)^23^. The pmf modulates the spatial organization of morphogenetic proteins^24^ as well as ATP homeostasis that is vital for bacterial survival^25^. These functions of the pmf offer an explanation for the effects observed with certain membrane-active compounds, albeit inhibition of which does not always result to cell death in many bacterial pathogens^26^. The potential use of salicylanilides as antimicrobials has drawn considerable interest as exemplified by recent studies demonstrating the anti-staphylococcal properties of closantel, niclosamide and oxyclozanide^27^,^28^. A limiting aspect is the low oral bioavailability of salicylanilides, which may render them ineffective in treating systemic infections. For instance, niclosamide was found to be only partially absorbed from the GI tract (with a maximal serum concentration ranging from 0.25 to 6 μg/mL after oral administration to human volunteers) and was also poorly distributed to tissues^29^. Closantel, rafoxanide and oxyclozanide exhibited similar pharmacokinetic (PK) attributes and were minimally metabolized and mostly excreted unchanged (up to ~90% for closantel) in the feces in ruminants^22^.

Despite the seemingly “unfavorable” PK profile, the salicylanilides may find niche therapeutic utility where poor GI absorption is desired. Herein, we provide evidence that the gut pathogen *C. difficile* makes for a viable target of such compounds. We show that the salicylanilide derivatives efficiently inhibited the growth of *C. difficile* via membrane depolarization, and more importantly, killed both logarithmic- and stationary-phase cells in a concentration-dependent manner. The bactericidal property against stationary-phase *C. difficile* could in principle lower the production of toxins and spores, which may in turn lead to improved response and mitigate CDI recurrence.

## Results and Discussion

### *In vitro* susceptibilities of *C. difficile* isolates

Over the last decade, *C. difficile* ribotype 027 was the predominant ribotype found in CDI fecal samples across North America and Europe^30^, however in recent years, other ribotypes are becoming increasingly prevalent^31^. We evaluated sixteen *C. difficile* clinical isolates of defined PCR ribotype (including the more prevalent ribotypes 027, 002, 014, 020, 078 and 106)^31^ for their *in vitro* susceptibilities to anthelmintics closantel, rafoxanide, niclosamide and oxyclozanide (Supplementary Table S1). The salicylanilides displayed broad activity against *C. difficile*, with MIC as low as 0.06–0.13 μg/mL for rafoxanide (Table 1). Based on the MIC against 16 isolates of *C difficile*, we determined the MIC_90_ values to be 0.25, 0.13, 2 and 1 μg/mL for closantel, rafoxanide, niclosamide and oxyclozanide, respectively (Table 1 and Supplementary Table S1). Metronidazole and vancomycin were also included as controls, which displayed MIC ranges of 0.13–0.25 μg/mL and 0.5–2 μg/mL, respectively, against the *C. difficile* isolates (Table 1 and Supplementary Table S1).

### Salicylanilides inhibit *C. difficile* growth via membrane depolarization

**Figure 2:**
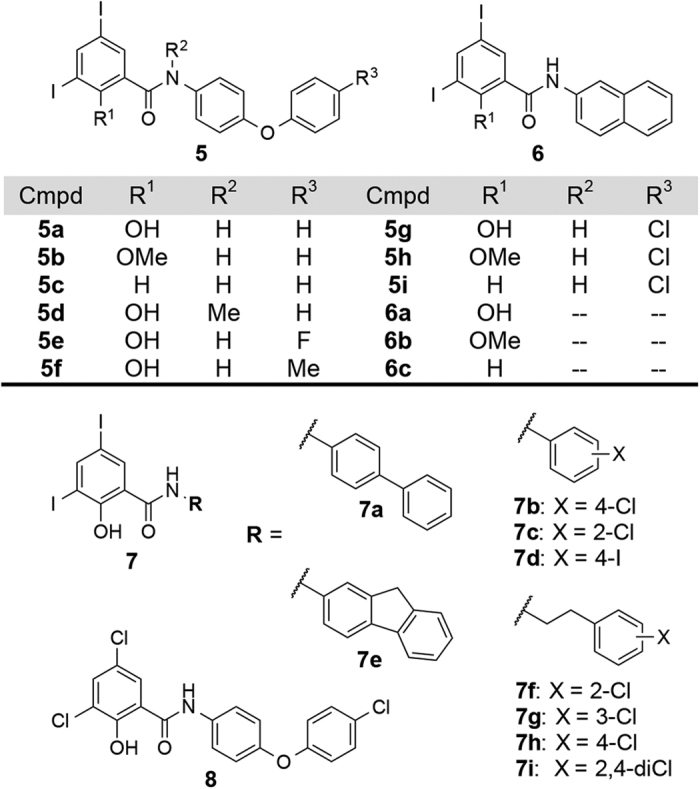
Structures of salicylanilide analogues.

In order to ascertain that the observed activity of the salicylanilides occurs through dissipation of the bacterial membrane potential, we prepared analogues **5** and **6** (Fig. 2) as previously described^32^, and evaluated their growth inhibitory activity against *C. difficile* strains 630 (CD630, ATCC BAA-1382-FZ, ribotype 012) and 4118 (CD4118, ATCC BAA-1870, ribotype 027). As a side note, CD630 is a virulent, multidrug-resistant strain whose genome has been completely sequenced^33^, while CD4118 is a BI/NAP1/027 hypervirulent pathogen. We have earlier delineated the structural features that are necessary for protonophoric activity of salicylanilides, requiring both a dissociable phenolic OH group and an amide proton that forms an intramolecular hydrogen bond to maintain hydrophobicity and stabilize the anionic form of the molecule (see Supplementary Fig. S1)^32^. The MIC values that were determined for **5** and **6** are consistent with a membrane depolarization mechanism as the compounds devoid of protonophoric activity [i.e. analogues that lack either the weakly acidic OH (**5b**, **5c**, **5h**, **5i, 6b** and **6c)** or the amide proton (**5d**)] were inactive, whereas protonophores **5a**, **5e**, **5f**, **5g**, and **6a** exhibited *in vitro* activity against CD630 and CD4118 (Table 2). Encouraged by these results, we explored several other derivatives, which harbor the diidosalicylate moiety coupled to varying substituents including biphenyl (**7a**), halogenated mono-aryl rings (**7b**–**d**), a fused-ring fluorenyl core (**7e**) and the more flexible ethylbenzenes (**7f**–**i**). Compounds **7a**-**i** demonstrated low MIC values (≤2 μg/mL), except for the *ortho*-chloro analogue **7c**, which showed reduced *in vitro* activity against CD630 and CD4118 (MIC = 8 μg/mL). Replacement of the diiodosalicylate with its dichloro congener **8** resulted in a 4-fold decrease in MIC relative to **5g** (Table 2). Compound **8** was also active against 14 other *C. difficile* isolates, with MIC values ranging from 0.03 to 0.25 μg/mL (Supplementary Table S1). The foregoing observations led us to probe other ionophores such as tropolones **9a**,**b**^34^ and β-carbolines **10a**,**b**^35^, as well as other structurally related compounds lacking the salicylanilide moiety (compounds **11a**–**f** and **12**, Supplementary Fig. S2); however, none of these were found to be active against CD630 and CD4118 (MIC > 32 μg/mL, Supplementary Table S2).

An attractive feature of membrane-active compounds is their low propensity for resistance^17^. In order to assess the potential for resistance development *in vitro*, we conducted serial passaging of CD4118 at sub-inhibitory concentrations of the salicylanilides. A total of 21 serial passages were performed and the results are shown in Supplementary Fig. S3. The changes in MICs after serial passages were minimal; final MICs of the salicylanilides were ≤4-fold higher than the initial (pre-passage) MICs, suggesting that emergence of resistance is likely to be low. The same held true for both metronidazole and vancomycin, with no substantial increase in MIC (~2-fold) even after 21 passages of CD4118 (Supplementary Fig. S3).

### Salicylanilides are bactericidal against logarithmic- and stationary-phase cultures

Although ionophores are known to dissipate the pmf that is crucial for bacterial energy metabolism, they do not always display bactericidal activity^26^,^28^. We were interested whether the salicylanilides possess cidal properties against stationary-phase *C. difficile*, because these cells are the primary producer of toxins and spores that contribute to *C. difficile* pathogenesis^4^. We selected the more active compounds (closantel, rafoxanide and **8**) and assayed them for minimum bactericidal concentration (MBC, defined as the lowest concentration of the antibacterial agent required to kill ≥99.9% of the initial inoculum) against growing and non-growing cells of the BI/NAP1/027 pathogen CD4118. As shown in Table 3, all three compounds displayed bactericidal activities against both logarithmic- and stationary-phase cells of CD4118 at concentrations close to their MIC values. The MBC_log_ values of the protonophores were determined to be 0.25–2 μg/mL (~4 to 8-fold greater than their respective MIC values). Importantly, the salicylanilides retained bactericidal activities against stationary-phase *C. difficile* cells, in stark contrast to metronidazole and vancomycin, which did not result in ≥3-log reduction of CD4118 cells at 32 μg/mL (Table 3).

**Figure 3:**
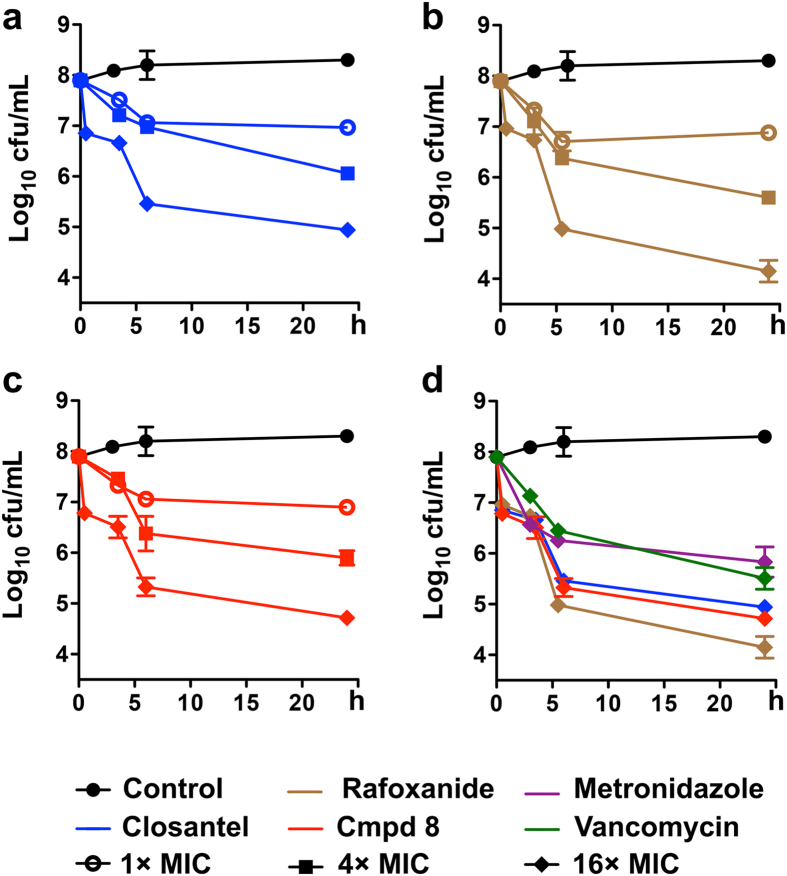
Time-kill kinetics against stationary-phase cultures of *C. difficile* strain 4118. Concentrations at 1× (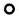), 4× (■) and 16× (♦) MIC of (**a**) closantel (**b**) rafoxanide, and (**c**) compound **8** are shown; (**d**) comparison of killing kinetics at 16× MIC of antimicrobials. Data plotted as mean log_10_ cfu/mL ± s.d. versus time in h (n = 2).

Next, we determined the time-kill kinetics of closantel, rafoxanide and **8** (at 1×, 4×, and 16× their respective MICs) against stationary-phase cultures of CD4118. As depicted in Fig. 3, all three salicylanilides showed a concentration-dependent mode of killing of the quiescent cells. At 16× the MIC of each protonophore, rafoxanide (at 2 μg/mL) eradicated >99.9% of viable cells in 6 h (Fig. 3b), while closantel (at 4 μg/mL) and compound **8** (at 1 μg/mL) achieved a similar activity in 24 h (Fig. 3a,c). At four-fold lower concentrations (i.e. 4 × MIC), rafoxanide caused a 2.7-log decrease in the number of CFUs in 24 h, comparable to those of closantel and **8**, which reduced bacterial cell viability by 2.2- and 2.4-log, respectively. In comparison, neither metronidazole (at 4 μg/mL) nor vancomycin (at 32 μg/mL) reached ≥3-log killing of CD4118 stationary-phase cells, even at 16× their respective MIC values (Fig. 3d). The rapid bactericidal property demonstrated by closantel, rafoxanide and **8** is an interesting finding because quiescent *C. difficile* cells are notoriously recalcitrant to antibiotic-mediated killing^16^. We surmise that the cidal effect of such protonophores on stationary-phase *C. difficile* cells would ameliorate the effect of toxin production and spore formation, similar to what was observed with other membrane-active compounds^16^.

### Antimicrobial spectrum of salicylanilides

In an effort to assess the antibacterial spectrum of the salicylanilides, we evaluated representative compounds (closantel, rafoxanide, **6a**, **7b**, **8**) against a panel of obligate aerobes, facultative anaerobes and obligate anaerobes. All five agents were generally more selective against Gram-positive bacteria, displaying *in vitro* growth inhibition of *B. subtilis* ATCC 6051, *S. aureus* RN4220 and *S. epidermidis* 1457 (MIC ≤ 0.25 μg/mL; Supplementary Table S3) and modest activity against other anaerobic clostridial species *C. sporogenes* ATCC 15579 and *C. clostridioforme* ATCC 25537 (MIC = 1–16 μg/mL). Interestingly, the salicylanilides were poorly active against *Bifidobacterium* and *Lactobacillus* species; their MIC values were ≥8 μg/mL against *B. breve* strain EX336960VC19 and *B. longum* subsp. *longum* strain 44B, whereas those against the *Lactobacillus* species (*L. johnsonnii* strain 135-1-CHN, *L. plantarum* ATCC 8014, *L. reuteri* strain CF48-3A) ranged from 16 to 32 μg/mL (Supplementary Table S3). Moreover, the compounds were ineffective against aerobic Gram-negative bacteria (MIC ≥ 32 μg/mL against *A. baumannii* M2 and *P. aeruginosa* PAO1) and had modest MIC values of ≥4 μg/mL against gut commensals *B. fragilis* CL05T00C42, *B. thetaiotaomicron* ATCC 29148, *P. distasonis* ATCC 8503 and *P. nigrescens* ATCC 33563. These results are consistent with those of niclosamide and oxyclozanide, which were shown to primarily target Gram-positive bacteria^28^. Compound **5i**, which does not possess protonophoric activity^32^, lacked antibacterial activity whereas metronidazole and vancomycin mainly targeted anaerobic bacteria and Gram-positive organisms, respectively (Supplementary Table S3). The complex multilayered cell envelopes of Gram-negative organisms impose a permeability barrier to antimicrobial agents and most likely account for the diminished activity observed for the salicylanilide molecules. Of note, rafoxanide had MIC values of ≤0.13 μg/mL for *C. difficile* (Supplementary Table S1), which rendered ≥32-fold selectivity over *Bifidobacterium*/*Lactobacillus* strains and Gram-negative gut commensals (*Bacteroides* species) that were tested (Supplementary Table S3). These results warrant further investigations in vivo to fully decipher the effect of the salicylanilides on the gut microbiome.

### *In vitro* cytotoxicity and hemolytic activity of salicylanilides

Although the salicylanilides have been used extensively in veterinary medicine, there is little information available concerning their biological effects on humans, except for niclosamide, which is FDA-approved for treatment of intestinal cestode infections. In order to gauge potential cytotoxicity of the salicylanilides, hemolysis using sheep erythrocytes and MTS^36^ assay using two human cell lines (liver carcinoma HepG2 and embryonic kidney HEK 293T/17) were performed. An important finding was that the salicylanilides (closantel, rafoxanide, niclosamide, oxyclozanide and compound **8**) did not cause rupture of red blood cells when tested at 32 μg/mL (Supplementary Fig. S4). However, treatment of human cell lines with niclosamide led to a marked decrease in viability even at a low concentration of 0.125 μg/mL (Supplementary Fig. S5). Despite its high *in vitro* cytotoxicity, niclosamide is considered a “safe drug” because of its minimal absorption from the GI tract and high plasma protein binding^29^, thus sparing the host cells from its uncoupling property. An intriguing observation was the comparably lower *in vitro* toxicities of compound **8** and the veterinary drugs (closantel, rafoxanide, oxyclozanide) toward HepG2 and HEK 293T/17 (Supplementary Fig. S5). For example, rafoxanide had no apparent effect on mammalian cell viability even at a concentration of 8 μg/mL, which is ≥64-fold higher than its MIC values against *C. difficile* (Supplementary Table S1). We note that these results do not guarantee drug safety (relative to niclosamide) but nevertheless indicate the potential for repositioning of the veterinary anthelmintics as human drugs.

A common cause of antibiotic failure is the inadequate penetration of the target infection site. In the case of CDI, it is imperative that the active drug achieves therapeutic levels in the colon to repress or eliminate the outgrowth of toxigenic *C. difficile*. This places the salicylanilide anthelmintics at a definite advantage; their low oral bioavailability and high fecal excretion (as observed in ruminants and humans)^22^,^29^ would in theory result in adequate gut concentrations necessary to disarm the target pathogen. A substantial feature of the salicylanilides (as we have shown for closantel, rafoxanide and **8**) is their bactericidal activity against stationary-phase cultures of hypervirulent *C. difficile*–a property that is not exhibited by many antibiotics including metronidazole and vancomycin^16^. Killing of hypervirulent stationary-phase *C. difficile* could likely suppress toxin production and sporulation, which in principle may lead to an improved sustained response and reduced recurrence rate. The clinical potential of membrane-active agents is demonstrated by daptomycin and telavancin, which function through permeabilization/depolarization of bacterial membranes and are FDA-approved to treat complicated skin and skin structure infections^37^,^38^. Our results exemplify notable attributes of membrane-active salicylanilide anthelmintics and demonstrate their potential for repurposing as anti-*Clostridium difficile* agents.

## Methods

### Bacterial strains

*Clostridium difficile* strain 630 (ATCC^®^ BAA-1382-FZ^TM^)*, Clostridium difficile* strain 4118 (ATCC^®^ BAA-1870^TM^), *Clostridium sporogenes* (ATCC^®^ 15579^TM^), *Clostridium clostridioforme* (ATCC^®^ 25537^TM^), *Bacteroides thetaiotaomicron* (ATCC^®^ 29148^TM^), *Parabacteroides distasonis* (ATCC^®^ 8503^TM^) *Prevotella nigrescens* (ATCC^®^ 33563^TM^), and *Bacillus subtilis* (ATCC^®^ 6051^TM^) were purchased from ATCC (Manassas, VA, USA). *Pseudomonas aeruginosa* PAO1 was provided by Dr. Kendra Rumbaugh. *Bacteroides fragilis* strain CL05T00C42, *Bifidobacterium longum* strain 44B, *Bifidobacterium* breve strain EX336960VC19, *Lactobacillus reuteri* strain CF48-3A, *Lactobacillus johnsonii* strain 135-1-CHN, and all other *C. difficile* isolates described in this study were from BEI Resources, NIAID, NIH.

### Determination of minimum inhibitory concentration (MIC)

MIC values were determined in cation-adjusted Mueller-Hinton broth (CAMHB) for bacteria that grow aerobically (*B. subtilis, S. aureus, S. epidermidis, A. baumannii*, *P. aeruginosa*) or in CAMHB supplemented with 5% laked horse blood for the *Lactobacillus* species, in accordance to CLSI guidelines^39^,^40^.

Bacteria that belong to the *B. fragilis* group (*B. fragilis, B. thetaiotaomicron*, *P. distasonis*), including the closely related *P. nigrescens*, were grown in Brucella broth supplemented with hemin (5 μg/mL), vitamin K_1_ (1 μg/mL) and 5% lysed horse blood, according to CLSI guidelines^41^. For growth of *Bifidobacterium* species, the broth used was lactic acid bacteria susceptibility medium (LSM, consisting of 90% v/v Iso-Sensitest broth and 10% v/v MRS broth) containing 0.03% L-cysteine, consistent with ISO guidelines^42^. MIC assays for all *Clostridium* species were performed in brain-heart infusion broth supplemented with 0.5% yeast extract (BHIS) containing 0.03% L-cysteine, as previously described^18^,^43^. Anaerobic bacterial cultures were performed in an anaerobic cabinet (Coy Lab Products Inc., Grass Lake, MI, USA) at 37 °C in a reducing anaerobic atmosphere (8% H_2_, 8% CO_2_, 84% N_2_). All broths and 96-well microtiter plates were pre-reduced (incubated anaerobically overnight) prior to use for anaerobic culture.

All MIC determinations were performed in 96-well microtiter plates using the broth microdilution method. A summary of bacterial growth medium and conditions is tabulated in the Supplementary Procedures. Briefly, two-fold serial dilutions of test compounds were inoculated with ~5 × 10^5^ cfu/mL bacteria. MIC was recorded as the lowest concentration of the test compound that inhibited visible bacterial growth after 20–24 h (or ~48 h for the *Lactobacillus* species) of incubation at 37 °C. MIC assays were performed in duplicate. MIC values were consistent between the two determinations; when slight variations were observed, MIC values were reported as MIC ranges.

### Serial passage experiments

*C. difficile* strain 4118 serial passages were performed in BHIS containing 0.03% L-cysteine, using the broth microdilution method. The MIC for each compound was first determined according to the procedure detailed above. After incubation for 20–24 h at 37 °C in an anaerobic chamber, the treated culture growing at 0.5 × MIC was used to inoculate the subsequent passage and this process was repeated a total of 21 times. Serial passage experiments were performed in duplicate.

### Determination of minimum bactericidal concentration (MBC)

The procedure for MBC determination was adapted from that of Alam *et al.*^44^. Briefly, a single overnight colony of *C. difficile* strain 4118 (streaked onto blood agar plate) was suspended in BHIS and grown to OD_600_ ~ 0.4–0.5 (logarithmic phase) or for 24 h (stationary phase). In 96-well microtiter plates, cultures were added to two-fold serial dilutions of test compounds in BHIS and incubated for 20–24 h at 37 °C in an anaerobic chamber. Viable counts were then enumerated on BHIS agar plates and the MBC was determined as the lowest concentration of the test compound that resulted in ≥3-log reduction of the initial cell inoculum, after incubation for 20–24 h at 37 °C. MBC measurements were performed in duplicate.

### Time-kill kinetics assay

The procedure for the killing rate assays was the same as described for MBC determination except that treated cultures were enumerated on BHIS agar plates at specific time points (t = 0, 3, 6 and 24 h). Briefly, stationary-phase cultures of *C. difficile* strain 4118 (prepared as described above) were treated with closantel, rafoxanide, compound **8** at 1×, 4×, 16× MIC or with metronidazole and vancomycin at 16× MIC. At t = 0, 3, 6 and 24 h, sample aliquots were withdrawn from the treated cultures and bacterial viability was scored by plating serial dilutions on BHIS agar plates. The agar plates were incubated in the anaerobic chamber for 20–24 h at 37 °C. Kinetic experiments were performed at least twice.

### *In vitro* cytotoxicity assay

Cell lines Hep G2 [HEPG2] (ATCC^®^ HB-8065^TM^) and 293T/17 [HEK 293T/17] (ATCC^®^ CRL-11268^TM^) were purchased from ATCC and cultured according to manufacturer’s instructions. HEPG2 or HEK 293T/17 cells were cultured in 96-well plates, and incubated at 37 °C in a 5% CO_2_ humidifying chamber for 24 h. Cells were then treated with test compounds at concentrations ranging from 0.125–32 μg/mL, and an MTS assay was performed at 16-h post-incubation at 37 °C in a 5% CO_2_ humidifying chamber, using the CellTiter 96 aqueous non-radioactive cell proliferation assay kit (Promega, Madison, WI, USA) per manufacturer’s instructions. MTS assays were performed in duplicate.

### Hemolysis assay

Sheep red blood cells (Innovative Research, Novi, MI, USA) were washed three times with PBS pH 7.4. A 3% cell suspension in PBS (100 μL) was added to test compounds in PBS (100 μL, to a final concentration of 32 μg/mL), and then incubated at 37 °C for 1 h. The plate was centrifuged at 500 × *g* for 10 min, and supernatants (100 μL) were transferred to a clean 96-well plate. Hemolysis was determined by measuring absorbance at 540 nm, with 1% Triton X-100 as the positive control and 0.5% DMSO in PBS as the negative control. Hemolysis assays were performed in triplicate.

## Additional Information

**How to cite this article**: Gooyit, M. and Janda, K. D. Reprofiled anthelmintics abate hypervirulent stationary-phase *Clostridium difficile. Sci. Rep.* **6**, 33642; doi: 10.1038/srep33642 (2016).

## Supplementary Material

Supplementary Information

## Figures and Tables

**Figure 1:**
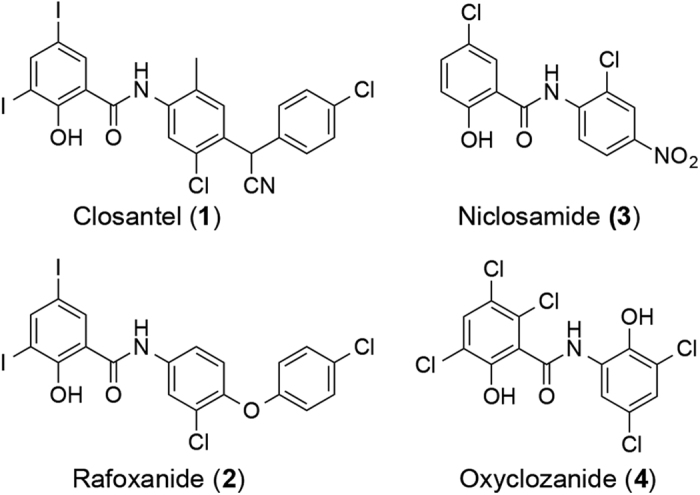
Structures of salicylanilide anthelmintics.

**Table 1:** *In vitro* susceptibilities of 16 *C. difficile* isolates.

Cmpd	MIC_50_	MIC_90_	MIC Range
Closantel	0.25	0.25	0.13–1
Rafoxanide	0.06	0.13	0.03–0.13
Niclosamide	1	2	0.5–4
Oxyclozanide	1	1	0.5–2
Metronidazole	0.25	0.25	0.13–0.25
Vancomycin	1	2	0.5–2

Abbreviations: MIC, minimum inhibitory concentration; MIC_50_ and MIC_90_, MIC at which 50% and 90% of the isolates are inhibited. All MIC values are expressed in μg/mL (n = 2).

**Table 2:** MIC values of salicylanilide analogues against *C. difficile* strains 630 and 4118.

Compound	MIC	
	*C. difficile* 630	*C. difficile* 4118
**5a**	0.5	1
**5b**	>32	>32
**5c**	>32	>32
**5d**	>32	>32
**5e**	0.13	0.13
**5f**	0.13	0.25
**5g**	0.13	0.25
**5h**	>32	>32
**5i**	>32	>32
**6a**	0.5	0.5
**6b**	>32	>32
**6c**	>32	>32
**7a**	0.5	1
**7b**	0.25	0.5
**7c**	8	8
**7d**	0.13	0.25
**7e**	0.25	0.5
**7f**	2	2
**7g**	1	1
**7h**	0.25	0.5
**7i**	0.25	0.5
**8**	0.03	0.06

All minimum inhibitory concentration (MIC) values are expressed in μg/mL (n = 2).

**Table 3:** *In vitro* activity against *C. difficile* strain 4118.

Cmpd	MIC	MBC_log_	MBC_stat_
Closantel	0.25	2	4
Rafoxanide	0.13	0.5	1
**8**	0.06	0.25	1
Metronidazole	0.25	>32	>32
Vancomycin	2	8	>32

Abbreviations: MIC, minimum inhibitory concentration; MBC_log_, minimum bactericidal concentration for logarithmic-phase cells; MBC_stat_, minimum bactericidal concentration for stationary-phase cells. All MIC and MBC values are expressed in μg/mL (n = 2).
