# Supplementary material for: Reprofiled anthelmintics abate hypervirulent stationary-phase Clostridium difficile
Source: Sci Rep. 2016 Sep 16;6:33642. doi: 10.1038/srep33642 (PMC5025651; doi:10.1038/srep33642)
Supplement: Supplementary Information [file srep33642-s1.pdf]

Reprofiled anthelmintics abate hypervirulent stationary-phase *Clostridium difficile*

Major Gooyit and Kim D. Janda*

Departments of Chemistry and Immunology and Microbial Science, The Skaggs Institute for Chemical Biology, and The Worm Institute of Research and Medicine, The Scripps Research Institute, 10550 North Torrey Pines Road, La Jolla, California 92037, United States

* Email: kdjanda@scripps.edu

Table of Contents

I. Supplementary Figures……………………………………………………………………………….S2
II. Supplementary Tables……………………………………………………………………………...S4
III. Supplementary Procedures……………………………………………………………………….....S6
IV. Supplementary References……………………………………………………………………….....S7

I. Supplementary Figures

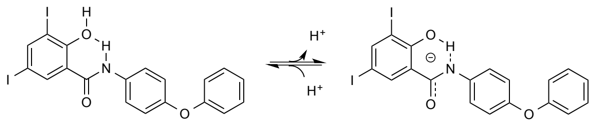

Figure S1. Model of protonophoric activity showing the neutral and anionic forms of closantel analogue **5a**. Adapted from Terada *et al*.[1]

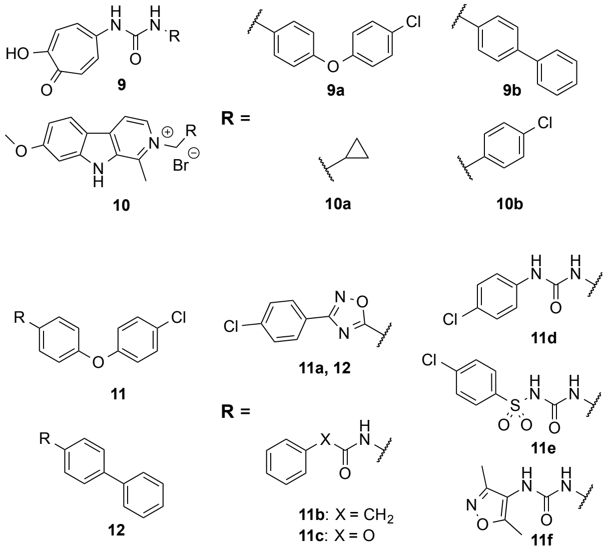

Figure S2. Structures of tropolones, β-carbolines and related compounds devoid of the salicylanilide moiety.

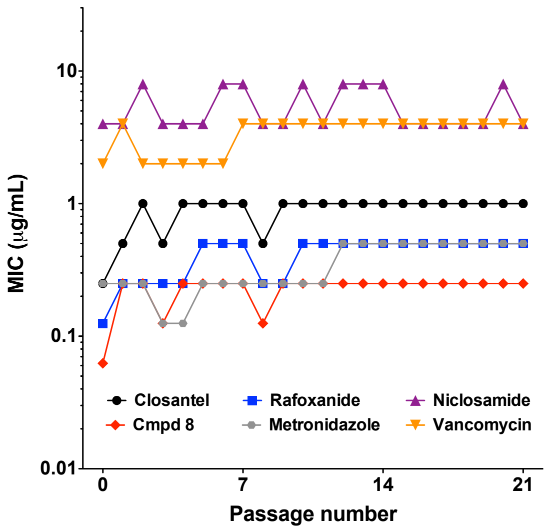

Figure S3. MIC values against *C. difficile* strain 4118 after the number of serial passages indicated

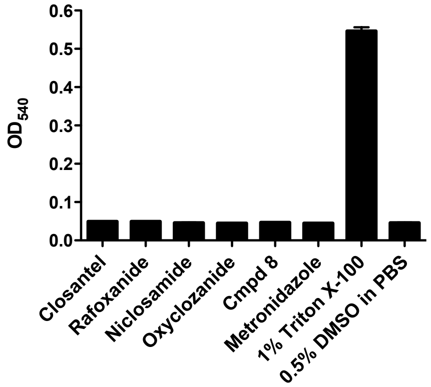

Cmpd	Hemolysis (%)[a]
Closantel	< 1
Rafoxanide	< 1
Niclosamide	< 1
Oxyclozanide	< 1
**8**	< 1
Metronidazole	< 1

[a]Determined at a concentration of 32 μg/mL using 1% Triton X-100 and 0.5% DMSO in PBS as the positive and negative controls, respectively. Assay performed in triplicate.

Figure S4. Hemolytic activity of select compounds.

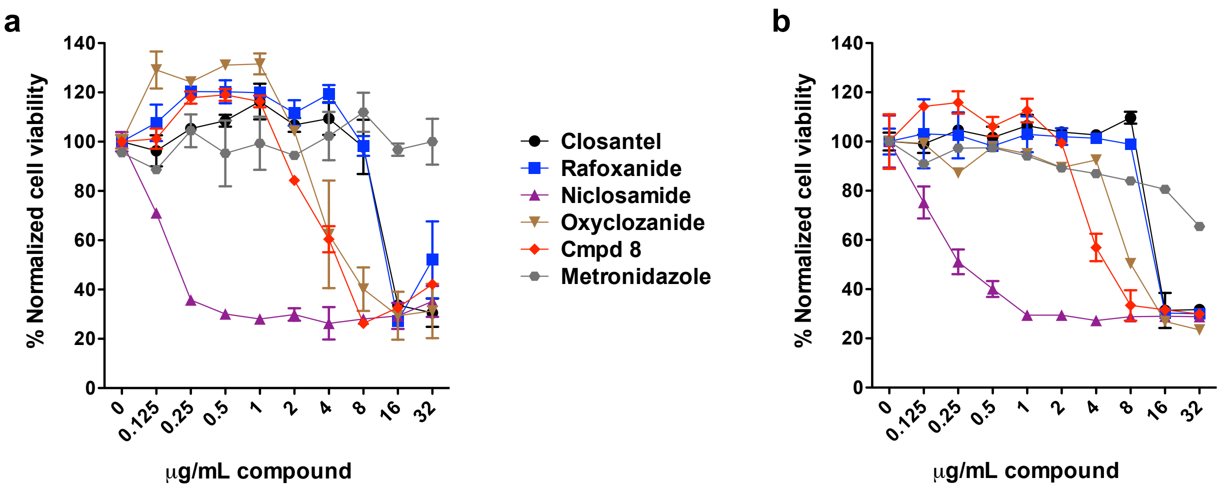

Figure S5. *In vitro* toxicity to (a) HEK 293T/17 and (b) HepG2 cells. Cell viability was evaluated after treatment with two-fold serially diluted concentrations (0.125 to 32 µg/mL) of test compounds, using the MTS assay.

II. Supplementary Tables

Table S1. *In vitro* susceptibilities of 16 *C. difficile* isolates

Clostridium difficile	Ribotype	MIC[a] (µg/mL)						
		Closantel	Rafoxanide	Niclosamide	Oxyclozanide	Cmpd **8**	Metronidazole	Vancomycin
ATCC BAA-1382-FZ (CD630)	012	0.13	0.06	1	0.5	0.03	0.25	1
ATCC BAA-1870 (CD4118)	027	0.25	0.13	4	1	0.06	0.25	2
Isolates[b]								
20110869	001_072	1	0.13	2	0.5−1	0.06−0.13	0.25	1
20110742	002	0.13−0.25	0.06	1	1	0.06	0.13	0.5−1
20100584	014	0.25	0.06	0.5	1	0.06	0.25	0.5
20110960	017	0.13	0.06	1	0.5	0.06	0.25	0.5
20110740	018	0.25	0.06	1	1	0.06	0.25	1
20120016	019	0.25	0.13	0.5	0.5	0.13	0.25	0.5
20110566	020	0.25	0.06	1	1	0.06	0.25	0.5
20111163	024	0.13−0.25	0.06	1	0.5	0.13	0.13	1
20121412	047	0.125	0.03	1	2	0.13	0.25	1
20100422	054	0.25	0.13	0.5	1	0.25	0.25	1
20110986	078	0.25	0.06−0.13	1	1	0.06	0.13−0.25	0.5−1
20110973	106	0.25	0.06	1	1	0.03	0.25	2
20121190	126	0.25	0.13	0.5	1	0.25	0.13	0.5
20110961	A12	0.25	0.06	1	1	0.13	0.25	1
MIC_{50}		0.25	0.06	1	1	0.06	0.25	1
MIC_{90}		0.25	0.13	2	1	0.25	0.25	2

[a]Performed in duplicate
[b]Obtained from BEI Resources, NIAID, NIH

Table S2. *In vitro* activity of compounds **9-12** against *C. difficile*

Cmpd	MIC[a] (µg/mL)	
	CD630 (ATCC BAA-1382-FZ)	CD4118 (ATCC BAA-1870)
**9a**	> 32	> 32
**9b**	> 32	> 32
**10a**	> 32	> 32
**10b**	> 32	> 32
**11a**	> 32	> 32
**11b**	> 32	> 32
**11c**	> 32	> 32
**11d**	> 32	> 32
**11e**	> 32	> 32
**11f**	> 32	> 32
**12**	> 32	> 32

[a]Performed in duplicate

Table S3. *In vitro* activity against select obligate/facultative aerobic and obligate anaerobic bacteria

strain	MIC[a] (µg/mL)							
	Closantel	Rafoxanide	**5i**	**6a**	**7b**	**8**	metronidazole	vancomycin
B. breve Strain EX336960VC19	> 32	16–32	> 32	32	8	8	> 32	0.5
B. longum subsp. *longum* Strain 44B	16	8	> 32	32	8	8	4–8	0.25
C. clostridioforme ATCC 25537	4	1	> 32	4	16	4	0.06	0.5
C. sporogenes ATCC 15579	1	1	> 32	8	16	4	0.25	2
L. johnsonii Strain 135-1-CHN	32	32	> 32	32	32	32	> 32	0.5
L. plantarum ATCC 8014	> 32	32	> 32	> 32	32	16	> 32	> 32
L. reuteri Strain CF48-3A	32	16	> 32	32	32	16	> 32	> 32
B. fragilis Strain CL05T00C42	> 32	32	> 32	32	32	16	0.5	32
B. thetaiotaomicron ATCC 29148	> 32	> 32	> 32	> 32	> 32	> 32	1	> 32
P. distasonis ATCC 8503	16	8	> 32	16	8	4	2	> 32
P. nigrescens ATCC 33563	8	8	> 32	8	4	4	2	> 32
B. subtilis ATCC 6051	≤ 0.03	≤ 0.03	> 32	0.06	0.06	≤ 0.03	> 32	0.13
S. aureus RN4220	0.25	0.25	> 32	0.25	0.06	0.13	> 32	1
S. epidermidis 1457	≤ 0.03	≤ 0.03	> 32	0.06	≤ 0.03	≤ 0.03	> 32	2
A. baumannii M2	> 32	> 32	> 32	> 32	32	32	> 32	> 32
P. aeruginosa PAO1	> 32	> 32	> 32	> 32	> 32	> 32	> 32	> 32

[a]Performed in duplicate. For clarity, MIC values against Gram-positive and Gram-negative bacteria are shown in blue and red, respectively.

III. Supplementary Procedures

A. Conditions for Bacterial Growth and MIC Determination

Bacteria	Agar plate	Growth medium	Growth conditions
All *Clostridium* species described in this study	Tryptic soy agar (TSA) with sheep blood (5%) or Blood agar base II with sheep blood (5%)	Brain-heart infusion broth with yeast extract (0.5%) and L-cysteine (0.03 %)	Anaerobic atmosphere (8% H_2, 8% CO_2, 84% N_2) at 37 °C; 20−24 h incubation time
B. breve EX336960VC19<br>*B. longum* subsp. *longum* 44B	TSA with sheep blood (5%)	(9:1) Iso-Sensitest/MRS broth with L-cysteine (0.03 %)	Anaerobic atmosphere at 37 °C; 20−24 h incubation time
L. johnsonii 135-1-CHN<br>*L. plantarum* ATCC 8014<br>*L. reuteri* CF48-3A	Lactobacilli MRS agar	Cation-adjusted Mueller Hinton broth supplemented with laked horse blood (5%)	Anaerobic atmosphere at 37 °C [5% CO_2 atmosphere (for *L. plantarum*)]; ~48 h incubation time
B. fragilis CL05T00C42<br>*B. thetaiotaomicron* ATCC 29148<br>*P. distasonis* ATCC 8503<br>*P. nigrescens* ATCC 33563	TSA with sheep blood (5%)	Brucella broth with hemin (5 µg/mL), Vitamin K_1 (1 µg/mL), and lysed horse blood (5%)	Anaerobic atmosphere at 37 °C; 20−24 h incubation time
B. subtilis ATCC 6051<br>*S. aureus* RN4220<br>*S. epidermidis* 1457<br>*A. baumannii* M2<br>*P. aeruginosa* PAO1	Cation-adjusted Mueller-Hinton agar	Cation-adjusted Mueller-Hinton broth	Aerobic atmosphere at 37 °C; 20−24 h incubation time

B. Synthesis and Characterization of Compounds

Closantel (Sigma), rafoxanide (TCI America), niclosamide (Combi-Blocks), oxyclozanide (Sigma), metronidazole (Combi-Blocks), and vancomycin hydrochloride hydrate (Sigma) were used as received.

Compounds **5a-i**, **6a-c**, **7a-d**, **9a-b**, **10a-b**, **11a-f** and **12** were prepared as previously described.[2-4] Compounds **7e-i** and **8** were synthesized according to published procedure.[2] Briefly, 3,5-diiodosalicylic acid (or 3,5-dichlorosalicylic acid, 1 eq) was heated to reflux with $SOCl_2$ (5 eq) for 7 h, and thereafter concentrated under reduced pressure. The corresponding acyl chloride product was precipitated with cold hexanes, filtered and air-dried. Coupling with the respective amine (1 eq) was performed in DMF in the presence of DIPEA (3 eq) at rt for 1 h. All salicylanilide products were purified by preparative HPLC.

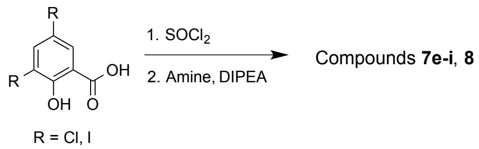

^{1}H and ^{13}C NMR spectra were recorded on Bruker DRX-600 equipped with a 5 mm DCH cryoprobe. Purity of all tested products were generally > 95% as assessed by HPLC.

N-(9*H*-Fluoren-2-yl)-2-hydroxy-3,5-diiodobenzamide (**7e**). Yield: 40%. ^{1}H NMR (600 MHz, $CDCl_3$) δ 3.94 (s, 2H), 7.30 − 7.34 (m, 1H), 7.39 (t, J = 7.3 Hz, 1H), 7.44 − 7.48 (m, 1H), 7.56 (d, J = 7.4 Hz, 1H), 7.75 − 7.81 (m, 2H), 7.81 (d, J = 1.8 Hz, 1H), 7.89 (s, 1H), 7.98 (s, 1H), 8.20 (d, J = 1.8 Hz, 1H). ^{13}C NMR (151 MHz, $CDCl_3$) δ 37.2, 80.4, 89.1, 116.9, 118.5, 120.0, 120.3, 120.5, 125.2, 127.0, 127.1, 134.3, 134.8, 139.9, 141.0, 143.4, 144.6, 151.1, 160.5, 166.4. HRMS-ESI (*m*/*z*): $[M + H]^+$ calcd for $C_{20}H_{14}I_2NO_2$, 553.9114; found, 553. 9110.

N-(2-Chlorophenethyl)-2-hydroxy-3,5-diiodobenzamide (**7f**). Yield: 49%. ^{1}H NMR (500 MHz, DMSO-d_6) δ 2.99 (t, *J* = 7.2 Hz, 2H), 3.54 (q, *J* = 7.0 Hz, 2H), 7.24 − 7.31 (m, 2H), 7.34 (dd, *J* = 2.1, 7.2 Hz, 1H), 7.44 (dd, *J* = 1.9, 7.3 Hz, 1H), 8.16 (d, *J* = 1.9 Hz, 1H), 8.18 (d, *J* = 1.9 Hz, 1H), 9.26 (t, *J* = 5.6 Hz, 1H). ^{13}C NMR (151 MHz, DMSO) δ 32.3, 81.4, 88.8, 116.2, 127.4, 128.4, 129.3, 131.2, 133.2, 135.1, 136.4, 149.4, 159.8, 168.1. HRMS-ESI (*m*/*z*): $[M + H]^+$ calcd for $C_{15}H_{13}ClI_2NO_2$, 527.8719; found, 527. 8706.

N-(3-Chlorophenethyl)-2-hydroxy-3,5-diiodobenzamide (**7g**). Yield: 53%. ^{1}H NMR (500 MHz, DMSO-d_6) δ 2.87 (t, *J* = 7.2 Hz, 2H), 3.53 (q, *J* = 7.0 Hz, 2H), 7.19 − 7.22 (m, 1H), 7.26 − 7.29 (m, 1H), 7.30 − 7.35 (m, 2H), 8.16 (d, *J* = 1.9 Hz, 1H), 8.17 (d, *J* = 1.9 Hz, 1H), 9.22 (t, *J* = 5.5 Hz, 1H). ^{13}C NMR (151 MHz, DMSO) δ 34.0, 40.5, 81.4, 88.9, 116.2, 126.3, 127.5, 128.6, 130.2, 133.0, 135.1, 141.6, 149.4, 159.8, 168.1. HRMS-ESI (*m*/*z*): $[M + H]^+$ calcd for $C_{15}H_{13}ClI_2NO_2$, 527.8719; found, 527. 8717.

N-(4-Chlorophenethyl)-2-hydroxy-3,5-diiodobenzamide (**7h**). Yield: 51%. ^{1}H NMR (500 MHz, DMSO-d_6) δ 2.85 (t, *J* = 7.2 Hz, 2H), 3.51 (q, *J* = 7.2 Hz, 2H), 7.27 (d, *J* = 8.5 Hz, 2H), 7.35 (d, *J* = 8.5 Hz, 2H), 8.16 (d, *J* = 1.9 Hz, 1H), 8.18 (d, *J* = 2.0 Hz, 1H), 9.21 (t, *J* = 5.4 Hz, 1H). ^{13}C NMR (151 MHz, DMSO) δ 33.7, 40.7, 81.4, 88.9, 116.2, 128.3, 130.6, 130.9, 135.1, 138.1, 149.4, 159.8, 168.0. HRMS-ESI (*m*/*z*): $[M + H]^+$ calcd for $C_{15}H_{13}ClI_2NO_2$, 527.8719; found, 527. 8713.

N-(2,4-Dichlorophenethyl)-2-hydroxy-3,5-diiodobenzamide (**7i**). Yield: 59%. ^{1}H NMR (500 MHz, DMSO-d_6) δ 2.97 (t, *J* = 7.0 Hz, 2H), 3.53 (q, *J* = 6.9 Hz, 2H), 7.35 − 7.39 (m, 2H), 7.60 (d, *J* = 1.1 Hz, 1H), 8.14 − 8.19 (m, 2H), 9.23 (t, *J* = 5.5 Hz, 1H). ^{13}C NMR (151 MHz, DMSO) δ 31.8, 81.4, 88.8, 116.2, 127.5, 128.7, 131.9, 132.5, 134.1, 135.1, 135.7, 149.4, 159.8, 168.2. HRMS-ESI (*m*/*z*): $[M + H]^+$ calcd for $C_{15}H_{12}Cl_2I_2NO_2$, 561.8329; found, 561. 8319.

3,5-Dichloro-*N*-(4-(4-chlorophenoxy)phenyl)-2-hydroxybenzamide (**8**). Yield: 55%. ^{1}H NMR (600 MHz, $CDCl_3$) δ 6.96 (d, *J* = 8.8 Hz, 2H), 7.04 (d, *J* = 8.9 Hz, 2H), 7.31 (d, *J* = 8.8 Hz, 2H), 7.50 (d, *J* = 2.3 Hz, 1H), 7.53 (d, *J* = 8.9 Hz, 2H), 7.56 (d, *J* = 2.3 Hz, 1H), 8.02 (s, 1H), 12.16 (s, 1H). ^{13}C NMR (151 MHz, $CDCl_3$) δ 116.6, 119.6, 120.3, 123.5, 123.7, 124.2, 124.6, 128.8, 130.0, 131.6, 134.3, 154.9, 155.8, 156.0, 166.6. HRMS-ESI (*m*/*z*): $[M + H]^+$ calcd for $C_{19}H_{13}Cl_3NO_3$, 407.9955; found, 407.9955.

IV. Supplementary References

(1) Terada, H.; Goto, S.; Yamamoto, K.; Takeuchi, I.; Hamada, Y.; Miyake, K. *Biochim. Biophys. Acta* **1988**, *936*, 504.
(2) Gooyit, M.; Tricoche, N.; Lustigman, S.; Janda, K. D. *J. Med. Chem.* **2014**, *57*, 5792.
(3) Gooyit, M.; Tricoche, N.; Javor, S.; Lustigman, S.; Janda, K. D. *ACS Med. Chem. Lett.* **2015**, *6*, 339.
(4) Gooyit, M.; Harris, T. L.; Tricoche, N.; Javor, S.; Lustigman, S.; Janda, K. D. *ACS Infect. Dis.* **2015**, *1*, 198.
